# Self-Designed Ningxin Anshen Formula for Treatment of Post-ischemic Stroke Insomnia: A Randomized Controlled Trial

**DOI:** 10.3389/fneur.2020.537402

**Published:** 2020-11-09

**Authors:** Ning Dai, Yuanyuan Li, Jing Sun, Feng Li, Hang Xiong

**Affiliations:** ^1^College of Traditional Chinese Medicine, Beijing University of Traditional Chinese Medicine (TCM), Beijing, China; ^2^Department of Acupuncture and Moxibustion, Dongfang Hospital, Beijing University of Traditional Chinese Medicine (TCM), Beijing, China; ^3^Department of Medical Neurology, Tongzhou District of Dongzhimen Hospital, Beijing University of Traditional Chinese Medicine (TCM), Beijing, China

**Keywords:** Ningxin Anshen Formula, post-stroke insomnia, Pittsburgh Sleep Quality Index, Insomnia Severity Index, randomized controlled trial

## Abstract

This study aimed to assess the efficacy and safety of self-designed Ningxin Anshen (NXAS) Formula for post-ischemic stroke insomnia of blood-deficient and liver-heat syndrome. Ninety patients were randomized into NXAS group, Placebo group and Zopiclone group. Patients in the NXAS group, Placebo group and Zopiclone group were treated with Ningxin Anshen Formula, placebo and zopiclone for 4 weeks, respectively. The scores of the Pittsburgh Sleep Quality Index (PSQI), Insomnia Severity Index (ISI) and traditional Chinese Medicine (TCM) Syndromes of self-designed scale and the number of adverse events (AEs) were determined. Results showed that the overall effective rate in the NXAS group and Placebo group was 76.67 and 30.00%, respectively, showing significant difference (*P* < 0.01). There was no marked difference between Zopiclone group (80.00%) and NXAS group. In both NXAS group and Zopiclone group, the scores of PSQI, ISI, and TCM Syndromes of self-designed scale after 4-week treatment were significantly different from those before treatment (*P* < 0.01). After 4-week treatment, the scores of PSQI, ISI, and TCM Syndromes of self-designed score were comparable between NXAS group and Zopiclone group (*P* > 0.05). Only one patient in the NXAS group developed gastrointestinal discomfort, which resolved without treatment discontinuation. In conclusion, self-designed NXAS Formula is effective and safe and has little adverse effect in treating post-stroke insomnia of blood-deficient and liver-heat syndrome.

## Introduction

Insomnia, classically defined as the difficulty in falling into and maintaining sleep most of time at night over a period of time with daytime consequences (1). Sleep and circadian rhythm disruptions are potentially modifiable risk factors and also the consequences of ischemic stroke. Palomäki et al. (2) found that insomnia was a common complaint after ischemic stroke. Pre-clinical evidence suggests a direct effect of sleep and endogenous circadian rhythm dysfunctions on the lesion volume and post-stroke recovery (3). The prevalence of insomnia complaints after stroke ranges between 3.8 and 57% (3, 4). These findings are supported by the study of Glozier et al. (5): in patients with chronic insomnia (16%) after stroke, the rates of anxiety, disability, and depression increased by 3.31, 3.60, and 6.75-folds, respectively (5). Therefore, the effective treatment of post-stroke insomnia is of great significance for the post-stroke rehabilitation. Ischemic stroke is related to the “asthenia in origin and sthenia in superficiality,” and the core of “asthenia in origin” is the “blood deficiency” according to the theory of Traditional Chinese Medicine (TCM). Ischemic stroke is caused by the insufficient blood perfusion to the brain due to complete or partial vascular occlusion, which is consistent with the theory of TCM on stroke. According to the theory of Western medicine, treatments for ischemic stroke aim to facilitate the blood flow, increase the blood perfusion and recanalize the collateral circulation, which also target the “blood deficiency” based on the theory of TCM. According to the “Qi, Blood, Essence, and Body Fluid” theory (6), the liver is responsible for “blood storage and catharsis” among the vital organs of the human body. If the function of “blood storage” is damaged in the liver, the “catharsis” will become dysfunctional in the liver, resulting in the “stagnation of liver-QI” and subsequent “liver-heat syndrome.” Based on the theory of TCM, the good sleeping is dependent on the balance between Yin and Yang. The “Yin deficiency,” “Yang hyperactivity” and “Yang failing to enter Yin” are closely related to the pathogenesis of insomnia in TCM. Patients with ischemic stroke usually have “blood deficiency” and “Yin fluid consumption,” which may cause “hyperactivity of liver-Yang” and “Yang failing to enter Yin,” leading to insomnia.

Many insomnia patients resort to over-the-counter medication, Complementary and Alternative Medicine and self-help methods (7–9). Chinese Herbal Medicine is one of the most common treatments for insomnia in Chinese populations (10, 11) and its use is increasing in Western countries (12). TCM is an ancient medical practice with a unique diagnostic system. A key method of diagnosis is to differentiate the etiology and pathogenesis, which are usually determined by the comprehensive analysis of clinical symptoms and signs collected through inspection, auscultation, olfaction, interrogation and pulse palpation (13). These are also known as TCM Syndromes according to which clinicians choose suitable treatments. Recent systematic reviews of randomized-controlled trials suggest that Chinese herbal medicine is relatively safe and can effectively improve sleep quality and sleep parameters (14, 15).

In our previous study, results showed the self-designed Ningxin Anshen (NXAS) Decoction effectively alleviated generalized anxiety disorder (blood-deficient and liver-heat syndrome), and especially it improves the sleep quality (16). Therefore, this study was to explore the efficacy and safety of self-designed NXAS Decoction in the treatment of post-stroke insomnia, which may provide a new strategy for the clinical treatment of post-stroke insomnia. As NXAS Decoction was proposed as an alternative treatment, zopiclone and placebo were used as controls. We hypothesized that NXAS had a similar efficacy to zopiclone and a better efficacy than Placebo, but its safety is superior to zopiclone. In the present study, the therapeutic efficacy was assessed by using the scores of the Pittsburgh Sleep Quality Index (PSQI) and Insomnia Severity Index (ISI), and the adverse events (AEs) were also recorded for the assessment of safety.

## Materials and Methods

### Study Design

**Figure 1:**
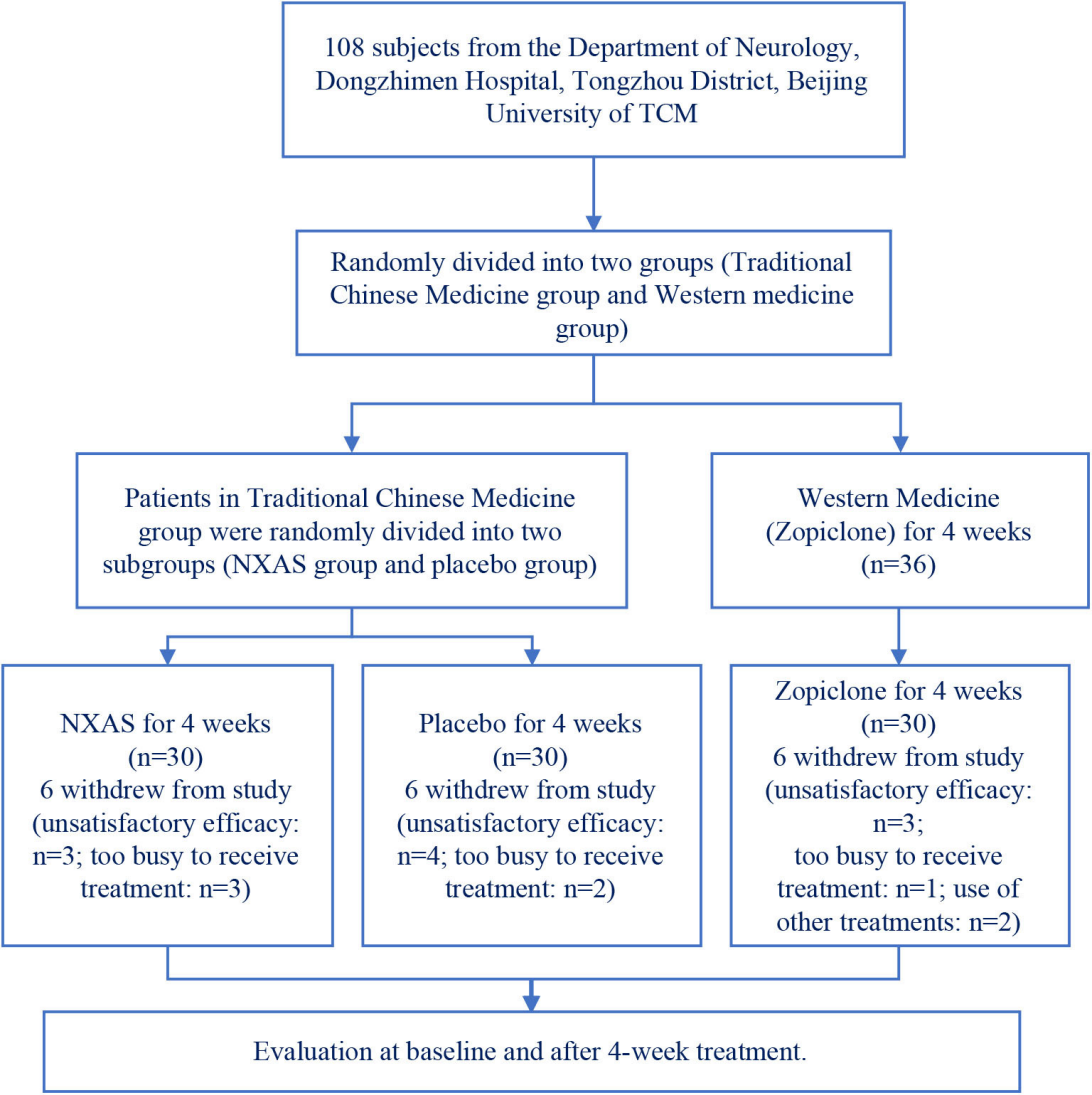
Study protocol of the randomized, controlled trial.

This study used a randomized, blinded, placebo-controlled study with 1:1:1 allocation ratio and parallel groups. The participants were randomized to the NXAS group, Placebo group or Zopiclone group and treated with NXAS Decoction, placebo, or zopiclone for 4 weeks. Patients were assessed before and after 4-week treatment. The study protocol is shown in Figure 1.

### Participant Recruitment Strategy

Participants were recruited between November 2017 and June 2019. The number of patients meeting the inclusion criteria was 108. The patients were randomly divided into three groups (*n* = 36 per group). Ten patients refused to continue to cooperate with the treatment due to unsatisfactory efficacy (four in Placebo group, three in NXAS group, and three in Zopiclone group). Five patients withdrew from this study because they were too busy to receive frequent follow up by hospital visit (three in NXAS group and two in Placebo group). One patient in the Zopiclone group could not be contacted during the follow up period. Two in the Zopiclone group took other drugs during the study period. No more than 20% of patients in each group was lost to follow up. Two patients took other Western medicine to treat sleep without permission, and not included for final analysis. Finally, only 90 patients were included for analysis (*n* = 30 per group). This study was registered with the China Clinical Trial Registry number ChiCTR1800015225.

### Screening Procedures

The potential participants received assessment before the first visit by telephone. At the first visit, the Neuropsychiatric Interview and the sleep disorders section of the Structured Clinical Interview for the Diagnostic and Statistical Manual of Mental Disorders (5th edition) were conducted by the principal investigator, who is a Chinese medicine practitioner trained in the diagnosis of mental disorders. In the first visit, the participant received routine blood test, liver function test, and electrolytes-urea-creatinine examinations (17).

The Stroke Impact Scale [SIS v.3; (18)] is a multidimensional measure of the impact of stroke on quality of life and often used to examine the changes of sleep quality after treatment. The SIS contains 59 items corresponding to eight subscales including: strength (S), hand function (HF), activities of daily living (ADL), mobility (M), communication (C), emotion (E), memory and thinking (MT), and social participation (SP). It is a 5-point Likert scale, and the score ranges from 1 to 5. Scores for each item were transformed using the following algorithm: [(actual raw score—lowest possible raw score)/possible raw score]×100. Higher scores signify better quality of life. In the present study, patients with SIS ≥ 75 were recruited aiming to exclude the influence of severity of stroke on the severity of insomnia (18).

Self-Rating Depression Scale (SDS) was developed by Shen et al. and is widely used to assess the depression. It contains 20 items and the feeling in prior week is scored with a 4-point system (1–4). The total score is calculated, and the standard score then determined. In Chinese population, no depression is defined as SDS score < 53. The scoring with Self-Rating Anxiety Scale (SAS) is similar to that of SDS. In Chinese population, no anxiety is defined as SAS <53 (19).

Self-designed TCM Syndrome Score and Diagnostic Basis of TCM: According to the diagnostic criteria for the insomnia in the Guidelines for Diagnosis and Treatment of Common Diseases in Internal Medicine of Traditional Chinese Medicine (20) and the National Standard for Terminology of Clinical Diagnosis and Treatment of Traditional Chinese Medicine (Syndrome Part) (21), the blood deficiency syndrome and liver-heat syndrome were confirmed. Blood deficiency syndrome refers to the loss of nourishment of viscera, meridians and body, with pale white or yellow complexion, light color of lips or tongue, dizziness, palpitation, numbness of hands and feet, pale color and others in TCM; the liver-heat syndrome is characterized by the stagnation of liver QI and the transformation of fire and has the common syndromes such as bitter mouth, chest and flank fullness, irritability and others. Eight main TCM syndromes were screened to quantify the disease, and a quantitative table of TCM syndromes for evaluating the efficacy of NXAS Formula in treating insomnia after ischemic stroke was preliminarily constructed. The major TCM symptoms include: (1) insomnia; (2) fatigue; the minor symptoms include: (1) dizziness; (2) plain faces; (3) flank pain or burning; (4) dreaming; (5) irritability; (6) dry or bitter mouth. Tongue: red or dark with yellow or thin and greasy moss. Pulse: string, fine or numbered. If the patient has two major symptoms and two minor symptoms, or insomnia is the main symptom and three minor symptoms appear, and the tongue and pulse signs match the characteristics, the patient can be diagnosed as blood deficiency syndrome and liver-heat syndrome. Each symptom was graded as follows: sever, 6; moderate, 4; mild, 2; asymptomatic, 0. Data of this scoring system were quantitative.

### Inclusion Criteria

The inclusion criteria, based on the diagnosis criteria for insomnia disorder (307.42) from the DSM5 (22), were as follows: (1) Patients were diagnosed with insomnia secondary to stroke by clinical manifestations and MRI or CT findings (ischemic stroke at the thalamus); (2) Patients had the characteristics of blood deficiency and liver-heat syndrome in TCM; (3) Patients were 35 to 75 years old; (4) Patients had difficulty in falling into sleep or maintaining sleep at least 3 times per week for at least 3 months despite adequate opportunity to sleep (23), which resulted in distress or impaired daytime functioning (usually present within 1 year after stroke); (5) the PSQI score was >7 and the ISI score was ≥10; (6) Patients did not receive any other treatment for insomnia, including pharmaceutical treatment, Complementary and Alternative Medicine, and psychotherapy at baseline and during the study period; (7) all participants were right-handed, the muscle strength of hemiplegic limbs was above grade 3, SIS score was ≥75 and they could cooperate with treatment and had no difficulty in urination and defecation; (8) An informed consent was obtained from each patient before study.

Exclusion criteria: (1) The blood examinations, including routine blood test, liver function test, and electrolytes-urea-creatinine examination, showed abnormalities within the past 6 months; (2) Patients received other treatments for insomnia within 14 days prior to randomization; (3) Patients had psychotic disorder, bipolar disorder, or other mental disorders such as depressive disorder or generalized anxiety disorder; (4) Patients had alcohol or drug addiction; (5) patients had cognitive impairment; (6) Patients were allergic to any of the ingredient of the NXAS Decoction, Placebo, or zopiclone; (7) Patients were treated with warfarin (24, 25), quetiapine, clozapine, or olanzapine (26); (8) Patients were pregnant or breast feeding women; (9) Patients were not suitable for other reasons determined by the investigators.

### Randomization and Treatments

The computer-generated random numbers were used for randomization by an investigator blind to this study. The participants were randomized during the first hospital visit. The trial unblinding was done in case of emergency, such as presence of serious adverse event that requires immediate treatment.

### Ethical Considerations

The study was approved by the Ethic Committee of Dongzhimen Hospital Affiliated to Beijing University of Chinese Medicine. All subjects provided written informed consent before study.

### Interventions

The investigated product was NXAS Decoction, a Chinese herbal medicine product composed of Suan Zao Ren (equivalent to 60 g of crude drug/package), Ci Wu Jia (equivalent to 45 g of crude drug/package), and Xia Ku Cao (equivalent to 10 g of crude drug/package). NXAS Decoction was extracted from these three kinds of crude traditional Chinese medicine and made into granule mixture in Beijing Kangrentang Pharmaceutical Co., Ltd. The placebo, composed of dextrin, had the same appearance to NXAS Decoction. The Placebo was also provided by Beijing Kangrentang Pharmaceutical Co., Ltd. In the Zopiclone group, zopiclone (Jilin Jinheng Pharmaceutical Co., Ltd., H20053718) was used. The participants in the NXAS group and Placebo group were orally treated with 1 bag of NXAS Formula and Placebo, respectively, twice daily (one in the morning and one in the evening) for 4 weeks. The participants in the Zopiclone group were orally treated with zopiclone (3.75 mg) once daily (before bedtime) for 4 weeks and 7.5 mg was administered if the therapeutic efficacy was unsatisfactory.

### Outcome Assessment

Baseline assessment: Demographic characteristics were collected, including age and gender. Vital signs (body temperature, pulse, respiration and blood pressure) were recorded. The liver function and renal function tests were done at baseline before randomization. The patients were assessed with self-rating anxiety scale and self-rating depression scale before treatment.

Primary Outcomes: The primary outcome was the changes in the PSQI and ISI scores after 4-week treatment as compared to those at baseline (week 0). The PSQI is a self-rated questionnaire consisting of 19 questions across 7 subscales (sleep quality, sleep latency, sleep duration, habitual sleep efficiency, sleep disturbance, use of hypnotics, and daytime dysfunction). Each subscale is scored on a scale of 0 to 3. Subscale scores are summed to a total score ranging from 0 (good sleep) to 21 (very poor sleep). The PSQI was verified as a reliable and valid measure of subjective sleep quality in clinical and experimental studies (27, 28). While the insomnia severity was measured with the Insomnia Severity Index. The ISI (29) is a brief 7-item scale used to assess the perceived insomnia severity and consequences in accordance with the diagnostic criteria for insomnia of the Diagnostic and Statistical manual of Mental Disorders (4th edition) and the International Classification of Sleep Disorders. Unlike the PSQI, the ISI is specific to insomnia disorder. The ISI has good psychometric properties (29) and can be used to assess the insomnia and evaluate the response to treatments (8). The score ranges from 0 to 28 and positively associated with insomnia severity.

Secondary Outcomes: The secondary outcomes included the scores of TCM Syndromes of self-designed scale and adverse events. The clinicians asked the participants to report the adverse events at each assessment. All the assessments were done at weeks 0 (baseline) and 4 (post-intervention). Different screenings were performed by a trained clinician blind to the study.

### Statistical Analysis

The quantitative data are expressed as mean ± standard deviation (SD) and compared with *T*-test or one-way ANOVA if normal distribution was present. Data with non-normal distribution are expressed as median ± interquartile range (IQR=P75-P25) and compared with non-parametric test. Qualitative data are expressed as rates and were compared with Chi-square test or non-parametric test. Statistical analysis was performed with SPSS version 20.0 by statisticians blind to the grouping. A value of *P* < 0.05 was considered statistically significant.

### Assessment of Therapeutic Efficacy

The therapeutic efficacy was assessed as follows: rehabilitation: the sleep time became normal or was longer than 6 h daily, sleep quality was good, and the mental state was good after waking up; significant improvement: the sleep quality was improved significantly, sleep time was longer than 3 h daily; Effectiveness: the sleep time increased, but was shorter than 3 h; Ineffectiveness: clinical symptoms remained unchanged or even worsened. Efficacy rate = (rehabilitation + significant improvement + Effectiveness)/total number of patients×100%.

## Results

### Demographics and Clinical Characteristics

The demographic characteristics are shown in Table 1. No significant differences were observed in the age, sex, education level, duration of post-stroke insomnia, SIS, SAS, and SDS scores among three groups at baseline. In addition, there were no significant differences in the behavioral features and adherence to sleep hygiene rules.

**Table 1:** Baseline characteristics (*n* = 90).

**Variable**	**NXAS (*n* = 30)**	**Zopiclone (*n* = 30)**	**Placebo (*n* = 30)**	***P*-value**
Female, *n* (%)	13 (43.33)	15(50.00)	15 (50.00)	0.837
Age(years), mean (SD)	59.87 (8.32)	60.47 (9.21)	59.10 (8.77)	0.817
Length of education (years), median (IQR)/mean (SD)	14.00 (3.50)^*^	14.87 (1.53)^#^	14.00 (4.00)^*^	0.526
Duration of PSI (months), mean (SD)	5.04 (2.43)	5.50 (2.61)	4.78 (2.76)	0.576
SIS, mean (SD)	80.77 (2.60)	80.67 (2.83)	79.93 (3.17)	0.417
SAS, median (IQR)	35.50 (9.00)	35.00 (8.00)	35.00 (8.00)	0.851
SDS, mean (SD)	33.60 (5.04)	33.70 (4.87)	33.20 (3.64)	0.904

*PSI, Post-stroke insomnia. SAS, self-rating anxiety scale; SDS, self-rating depression scale*;

* *median (IQR)*;

# *mean (SD)*.

### Primary Outcomes

#### Therapeutic Efficacy in Each Group

There was significant difference in the therapeutic efficacy among three groups (Pearson χ^2^ = 19.947, *P* < 0.01). There was no marked difference in the therapeutic efficacy between NXAS group and Zopiclone group (Pearson χ^2^ = 0.098, *P* = 0.754), but significant difference was noted between NXAS group and placebo group (Pearson χ^2^ = 13.125, *P* < 0.01) and between Zopiclone group and placebo group (Pearson χ^2^ = 15.152, *P* < 0.01) (Table 2).

**Table 2:** Comparison of clinical efficacy in each group.

**Variable**	**NXAS (*n* = 30)**	**Zopiclone (*n* = 30)**	**Placebo (*n* = 30)**
Rehabilitation, *n*	2	1	0
Markedly effective, *n*	10	11	0
Effective, *n*	11	12	9
Invalid, *n*	7	6	21
Effectiveness (%)	76.67	80.00	30.00
*P*-value		<0.01	

### PSQI and ISI Scores in Different Groups

As shown in Table 3, there were no significant differences in the scores of PSQI (*F* = 1.724, *P* = 0.184) and ISI (χ^2^ = 2.391, *P* = 0.303) among these groups before treatment. After 4-week treatment, the scores of PSQI (χ^2^ = 50.668, *P* < 0.01) and ISI (χ^2^ = 27.419, *P* < 0.01) in the NXAS group and Zopiclone group were markedly lower than in the Placebo group (*P* < 0.05) (Table 3). Moreover, there were no significant differences in the scores of PSQI (*P* = 0.629) and ISI (*P* = 0.136) between NXAS group and Zopiclone group after treatment (Table 3). In the Placebo group, the scores of PSQI (95% CI: −0.017–1.017, *t* = 1.980, *P* = 0.057) and ISI (95% CI: −0.665–3.132, *t* = 1.300, *P* = 0.199) remained unchanged after treatment.

**Table 3:** Scores of PSQI and ISI in 3 groups.

**Group (*n* = 30)**	**Before treatment**		**After treatment**	
	**PSQI mean (SD)**	**ISI median (IQR)**	**PSQI median (IQR)**	**ISI median (IQR)**
NXAS	13.20 (2.75)	17.00 (5.00)	7.00 (5.00)^*^	11.00 (6.00)^*^
Zopiclone	14.50 (2.87)	16.00 (5.00)	8.00 (2.25)^*^	9.50 (5.00)^*^
Placebo	14.23 (2.97)	17.00 (8.00)	13.50 (5.00)^Δ^	16.00 (6.00)^Δ^
*P*-value	0.184	0.303	<0.01	<0.01

*PSQI, Pittsburgh Sleep Quality Index; ISI, Insomnia Severity Index*.

* *P < 0.01: before vs. after*;

Δ *P > 0.05: before vs. after*.

### Secondary Outcomes

As shown in Table 4, there was no significant difference in the score of TCM Syndrome Scale (χ^2^ = 3.079, *P* = 0.214) among three groups before treatment. After 4-week treatment, there was significant difference in the score of TCM Syndrome Scale among three groups (χ^2^ = 16.648, *P* < 0.01). The TCM Syndrome Scale score remained unchanged in the Placebo group after treatment (*Z* = −1.820, *P* = 0.069). However, the score of TCM syndrome scale in the NXAS group (*Z* = −4.454) and Zopiclone group (*Z* = −4.775) was improved significantly after treatment (*P* < 0.01) (Table 4). After treatment, there was significant difference in the score of TCM syndrome scale between Placebo group and NXAS group (*Z* = −4.179)/Zopiclone group (*Z* = −4.392) (all *P* < 0.01). But there was no marked difference between NXAS group and the Zopiclone group (*Z* = −0.339, *P* = 0.735).

**Table 4:** Scores of TCM Syndromes of self-designed scale in 3 groups.

**Group (*n* = 30)**	**Pre-treatment median (IQR)**	**Post-treatment median (IQR)**
NXAS	14.00 (4.00)	8.00 (6.00)^*^
Zopiclone	16.00 (7.00)	8.00 (5.00)^*^
Placebo	14.00 (4.00)	12.00 (6.00)^Δ^
*P*-value	0.214	<0.01

* *P < 0.01: before vs. after*;

Δ *P > 0.05: before vs. after*.

In the Zopiclone group, fatigue and nausea were recorded in four and two patients, respectively, and dyspepsia was reported in one patient of NXAS group. These adverse effects resolved gradually without specific treatments. There was no adverse event in the Placebo group. There was a significant difference in the incidence of adverse events among three groups (χ^2^ = 9.604, *P* < 0.01). As shown in Table 5, there was no marked difference between NXAS group and Placebo group in the incidence of adverse events (χ^2^ = 1.017, *P* = 0.313). Moreover, after 4-week treatment, the body temperature, blood pressure, pulse, and findings from routine blood, urine, stool routine, liver and kidney function examinations were normal in three groups.

**Table 5:** Adverse events in 3 groups during treatment.

**Group (*n* = 30)**	**Adverse events *n* (%)**	**No adverse events *n* (%)**
NXAS	1 (3.3)	29 (96.7)
Zopiclone	6 (20.0)	24 (80.0)
Placebo	0 (0.0)	30 (100.0)
χ^2^	9.604	
*P*-value	0.008	

## Discussion

Insomnia is highly prevalent in stroke patients. It has been reported that the prevalence of insomnia is as high as 50% in the first month after stroke (30). The insomnia appears *de novo* in 1/3 of stroke patients; 2/3 of stroke patients have a history of insomnia before stroke. Post-stroke insomnia often results from environmental factors (such as light and noise in the stroke unit) or comorbidities (depression, pain and other diseases). Less commonly, insomnia may be directly related to brain injury. A recent meta-analysis indicates that short sleep, mostly defined as <5–6 h sleep/night, is an independent predictor of incident stroke after adjustment for age, sex and vascular risk factors (31). It has been suggested that insomnia symptoms may result in high indirect costs from loss of productivity (32). In experimental stroke models, sleep loss/deprivation was found to augment brain injury and impair neuroplasticity, whereas drugs that improve sleep exerted protective effects on the neuroplasticity and recovery after stroke (33).

However, few studies have been conducted to investigate the post-stroke insomnia (34). In a case control study, results showed stroke patients had significantly more problems in initiating sleep as compared to Zopiclone subjects (35). At the acute stage, a significant suppression of rapid eye movement (REM) sleep has been found in patients with ischemic hemispheral lesions, and after brain stem stroke, both REM and non-REM reduction are also observed (34). Recently, polysomnographic evidence has shown that the selective serotonin reuptake inhibitor citalopram alleviates the insomnia in a non-depressed stroke patient (36). In the present study, our results showed Placebo had no influence on the post-stroke insomnia, suggesting that post-stroke insomnia may be related to not only subjective condition, but also to stroke. In addition, the investigators and patients were blind to the treatment in the study, and thus the Hawthorne effect may not be obvious in our study. Currently, pharmacotherapy and psychotherapy are the only treatments for insomnia recommended by guidelines (37–40). Despite being the most common drugs for the treatment of insomnia (41), benzodiazepine receptor agonists have been found to have some adverse effects (38, 42), and there are also insomnia relapse, withdrawal symptoms (43), and concerns about dependence (44, 45). Unsurprisingly, most insomnia patients have a preference to nonpharmacological interventions (46, 47). Cognitive Behavioral Therapy for Insomnia (CBT-I), a set of psychotherapeutic interventions used specifically to treat insomnia disorder, has been proposed as an alternative. The efficacy of CBT-I for insomnia disorder is well-established (43); however, the lack of trained practitioners prevents most of insomnia patients benefiting from this therapy (48).

Because of less adverse effects and no addiction, traditional Chinese medicine has been a common alternative therapy in China. The traditional use of Chinese herbal medicine is often individualized based on the cause, pathogenesis, phase, phenotype, demographics, and comorbidities of insomnia patients (49). The present study was to assess the safety and efficacy of NXAS formula for post-stroke insomnia as compared to placebo and zopiclone. According to the theory of TCM, the pathogenesis of post-stroke insomnia of blood deficiency and liver-heat syndrome is related to the “stagnation of Qi and fire,” “loss of yin and blood,” and “restlessness of mind.” Blood deficiency syndrome refers to the loss of nourishment of viscera, meridians and body, which may lead to the loss of mind. This is also the cause of insomnia and thus we should nourish viscera, meridians and body in order to improve the symptoms of insomnia. Liver-heat syndrome is the stagnation of liver QI into fire. The stagnation of QI causes liver-heat, which disturbs the state of mind, thus causing insomnia. Thus, we should relieve QI stagnancy in the liver. The self-designed NXAS Prescription meets all the above requirements: the fried Suan-Zao-Ren (jujube seed or ziziphi spinosae semen) in the NXAS Prescription may function to nourish the heart, benefit liver and calm mind. NXAS Formula includes Suan-Zao-Ren granule, Ci-Wu-Jia granule and prunella vulgaris granules. Suan-Zao-Ren granule has been used to treat insomnia for many years (50), and Suan-Zao-Ren has been used for treatment of insomnia in China for centuries. Some studies have proposed that the most frequently used herb for the treatment of insomnia is Suan-Zao-Ren, and a number of herbal formulae for the treatment of insomnia are consistent with current clinical practice and have favorable prospects for further therapeutic development (49). There is evidence showing that Suan-Zao-Ren aqueous extract may ameliorate the symptoms of insomnia by modulating the levels of monoamines and amino acid neurotransmitters in the rat brain (51). Our previous study also revealed that the self-designed NXAS Prescription affected the synthesis and decomposition of glutamic acid (Glu) and γ-aminobutyric (GABA) and improve the excitatory/inhibitory metabolic imbalance of Glun/GABA, exerting therapeutic effects on the insomnia (52). The Suan-Zao-Ren has the largest dose in the NXAS formula, and thus we speculate that the Suan-Zao-Ren is mainly responsible for the therapeutic effects on the insomnia. In addition, Ci-Wu-Jia has also been found to decrease oxidative stress and inhibit apoptosis in impaired cells (53). Moreover, Ci-Wu-Jia can also significantly prolong the sleep time of mice treated with pentobarbital sodium (54). Another study indicated that Ci-Wu-Jia reduced the fragments of drosophila's sleep time and changed the sleep rhythm of Drosophila melanogaster under 24-h constant darkness (55). Prunella vulgaris with the function of clearing fire eyesight has been widely used in clinical practice. Prunella vulgaris mainly contains terpenoids, phenolic acids, flavonoids, sterols, coumarins, organic acids, volatile oils and carbohydrates, and other ingredients with significant sedative and hypnotic effects (56). Prunella vulgaris is widely used as an herbal medicine for the treatment of cancers, inflammatory diseases, and infections. Although it has long been used in clinical practice, few studies have examined its effects on the function of central nervous system. There is evidence showing that ethanolic extracts of Prunella vulgaris (EEPV) prolongs pentobarbital-induced sleep duration in insomnia mice (57). Taken together, the components of NXAS support the therapeutic effects of NXAS on insomnia.

In this prospective study, our results indicate that NXAS formula is safe and effective to improve the symptoms of insomnia in Chinese patients after stroke. The therapeutic efficacy of NXAS is similar to that of zopiclone, as measured by PSQI, ISI and TCM Syndromes of self-designed scores after 4-week treatment. Additionally, few adverse effects are recorded after NXAS treatment. Thus, NXAS formula supplementation may be used as an alternative to improve sleep quality in patients after ischemic stroke.

### Limitations

This study has the following limitations. First, patients were not followed up, which limits the generalizability of our conclusions. Second, only subjective measurements were done (PSQI, ISI) in the present study. But in the clinic, they are often used to assess the patient's sleep quality because of convenience. In our future studies, more objective tools (e.g., polysomnography) will be employed for the assessment of sleep quality, and the use of both subjective and objective measures are also recommended (58, 59). Third, according to the scores of PSQI and IS in each group before treatment (Table 3), the overall severity of insomnia in each group was not serious. Therefore, the regression mean may be not significant because it often happens when it is very serious. We would investigate this issue in future studies with large sample size. In addition, although all participants expressed that they didn't mind the drugs used (traditional Chinese medicine or Western Medicine), some still minded the treatments, for example, he/she preferred Western Medicine, but he/she was divided into TCM group, or *vice versa*. All these factors may affect the therapeutic efficacy. Previous studies have confirmed the therapeutic effects of jujube kernel, acanthopanax senticosus and prunella on the insomnia (60–62). Suan-Zao-Ren is the prince drug of NXAS formula, and saponin and flavonoids are the main active ingredients. It has been shown a correlation between jujuboside A and insomnia protein targets (63). Therefore, more studies are needed to investigate the mechanism underlying the effects of main components of self-designed NXAS formula in the treatment of insomnia.

## Conclusions

In conclusion, our results suggest that 4-week treatment with self-designed NXAS formula is effective and safe for post-stroke insomnia of blood-deficient and liver-heat syndrome.

## Data Availability Statement

The raw data supporting the conclusions of this article will be made available by the authors, without undue reservation.

## Ethics Statement

The studies involving human participants were reviewed and approved by Ethic Committee of Dongzhimen Hospital Affiliated to Beijing University of Chinese Medicine. The patients/participants provided their written informed consent to participate in this study.

## Author Contributions

ND and YL designed the work, collected and analyzed data, and participated in drafting the manuscript. JS and FL collected and analyzed data and revised the manuscript. HX contributed to the study design, and reviewed and revised the manuscript. All authors contributed to the article and approved the submitted version.

## Conflict of Interest

The authors declare that the research was conducted in the absence of any commercial or financial relationships that could be construed as a potential conflict of interest.
